# A Ca_V_2.1 N-terminal fragment relieves the dominant-negative inhibition by an Episodic ataxia 2 mutant

**DOI:** 10.1016/j.nbd.2016.05.020

**Published:** 2016-09

**Authors:** Shehrazade Dahimene, Karen M. Page, Manuela Nieto-Rostro, Wendy S. Pratt, Marianna D'Arco, Annette C. Dolphin

**Affiliations:** Department of Neuroscience, Physiology and Pharmacology, University College London, Gower Street, London WC1E 6BT, UK

**Keywords:** DRG, dorsal root ganglion, EA2, Episodic ataxia 2, FHM1, familial hemiplegic migraine type 1, SCA6, spinocerebellar ataxia type 6, TAT, transactivator of transcription, Episodic ataxia-2, P/Q-type calcium channel, Dominant negative suppression, N-terminus, Misfolded protein

## Abstract

Episodic ataxia 2 (EA2) is an autosomal dominant disorder caused by mutations in the gene *CACNA1A* that encodes the pore-forming Ca_V_2.1 calcium channel subunit. The majority of EA2 mutations reported so far are nonsense or deletion/insertion mutations predicted to form truncated proteins. Heterologous expression of wild-type Ca_V_2.1, together with truncated constructs that mimic EA2 mutants, significantly suppressed wild-type calcium channel function, indicating that the truncated protein produces a dominant-negative effect (Jouvenceau et al., 2001; Page et al., 2004). A similar finding has been shown for Ca_V_2.2 (Raghib et al., 2001). We show here that a highly conserved sequence in the cytoplasmic N-terminus is involved in this process, for both Ca_V_2.1 and Ca_V_2.2 channels. Additionally, we were able to interfere with the suppressive effect of an EA2 construct by mutating key N-terminal residues within it. We postulate that the N-terminus of the truncated channel plays an essential part in its interaction with the full-length Ca_V_2.1, which prevents the correct folding of the wild-type channel. In agreement with this, we were able to disrupt the interaction between EA2 and the full length channel by co-expressing a free N-terminal peptide.

## 1 Introduction

Ca_V_2.1 (P/Q-type) and Ca_V_2.2 (N-type) channels are voltage-gated calcium channels that are expressed in both the central and peripheral nervous system, where they are preferentially localized in presynaptic terminals and play a central role in neurotransmitter release (Turner et al., 1993). Mutations in the *CACNA1A* gene that encodes the pore-forming Ca_V_2.1 α1 subunit cause three neurological disorders: familial hemiplegic migraine type 1 (FHM1), spinocerebellar ataxia type 6 (SCA6) and Episodic ataxia 2 (EA2) (Pietrobon, 2010). EA2 is a rare autosomal dominant disorder characterized by prolonged episodes of ataxia, which are commonly triggered by emotional and physical stress (Jen, 2008). Interestingly, while all FHM1 mutations reported so far are missense mutations, localized to important functional regions of Ca_V_2.1 such as the pore and the voltage sensors, EA2 is frequently associated with nonsense, deletion or insertion mutations (Jeng et al., 2008, Mantuano et al., 2010). Indeed the majority of EA2 mutations described to date are predicted to form truncated proteins resulting from a premature stop codon (Pietrobon, 2010).

It has been found that the functional expression of the full-length Ca_V_2.1 channel is substantially suppressed when it is co-expressed with truncated constructs mimicking EA2 mutations (Jouvenceau et al., 2001, Page et al., 2004), indicating that EA2 may not be simply a result of haploinsufficiency. Furthermore, heterologous expression of the wild-type Ca_V_2.2 channel together with corresponding truncated constructs similarly suppressed wild-type channel function (Raghib et al., 2001). Our evidence suggests that the truncated proteins are recognized as misfolded proteins, and retained in the endoplasmic reticulum where they trigger endoplasmic reticulum stress (Page et al., 2004), and are also targeted for proteasomal degradation (Mezghrani et al., 2008). Furthermore, the suppression effect requires interaction between the full-length and the mutant protein, to induce both synthesis arrest and channel degradation, thereby reducing functional expression of the full-length channel (Mezghrani et al., 2008, Page et al., 2004).

Strikingly, the suppressive effect mediated by the truncated channel proteins has also been described for other calcium channels and may play a physiological role in regulating current density. Indeed, two-domain truncated forms of Ca_V_1.2 channel have been identified. These splice variants are predominantly expressed in fetal and neonatal rat heart (Wielowieyski et al., 2001). Furthermore a truncated two domain form of Ca_V_2.1 has been identified to occur in brain (Arikkath et al., 2002). Moreover, a truncated Ca_V_1.3 splice variant, Cav1.3 33 L, consisting of Dom I, II, III and a portion of domain IV, affects the function of the full-length channel (Liao et al., 2015). Thus, the suppressive effect of the truncated protein appears to play a physiological role in regulating Ca_V_1.3 function during cardiac development (Liao et al., 2015). Recently, Ca_V_1.2 was also shown to undergo proteolytic cleavage resulting in two complementary fragments. This mid-channel proteolysis is described as an activity-dependent feedback inhibition of voltage-dependent calcium channels (Michailidis et al., 2014).

For both Ca_V_2.1 and Ca_V_2.2 it has been shown that a motif in the N-terminus plays an important role in channel function and modulation by second messengers (Page et al., 1998). Initially, it was established that the substitution of just two arginine residues in this motif completely abolished G-protein modulation (Canti et al., 1999). Later, this motif was also found to be essential for the process underlying dominant-negative suppression of Ca_V_2.1 and Ca_V_2.2 currents (Page et al., 2010).

In this study we wished to explore whether overexpressing these key N-terminal residues as a separate peptide would impede the dominant-negative effect of the truncated EA2 protein, and thus restore the function of the wild-type Ca_V_2.1 channels. If so, this would provide a potential route towards therapeutic intervention.

## 2 Materials and methods

### 2.1 Molecular biology and constructs

The following cDNAs were used: rabbit Ca_V_2.2 HA (Cassidy et al., 2014), rat Ca_V_2.1 (GenBank Accession number M64373 with E1686R mutation) (Page et al., 2004), rat α_2_δ-1 (GenBank Accession number M86621), rat β1b (Tomlinson et al., 1993), Ca_V_2.2 Dom I–II (Raghib et al., 2001), EA2 mutant Ca_V_2.1-P1217fs (Page et al., 2004) Ca_V_3.1 Dom I–II (Page et al., 2004), and GFP-CAAX (Page et al., 2010). Other cDNAs were made using standard cloning techniques as follows: Ca_V_2.2^R52A, R54A^ HA, corresponding to the full-length Ca_V_2.2 contains R52A, R54A mutations and an exofacial HA tag. Ca_V_2.2 Dom I–II^R52A, R54A^ corresponds to the truncated Ca_V_2.2 with R52A, R54A mutations. Ca_V_2.1^R57A, R59A^ and EA2^R57A, R59A^ corresponds to the full-length and truncated Ca_V_2.1 with R52A, R54A mutations. For Ca_V_2.2 Dom I–II HA and Ca_V_2.2 Dom I–II^R52A, R54A^ HA, the exofacial HA tag was inserted as in Ca_V_2.2 HA (Cassidy et al., 2014). Ca_V_2.1-(46-100)-CAAX and Ca_V_2.2-(43-95)-CAAX correspond to the truncated N-terminal constructs, in which a CAAX motif was added to the C-terminus (Page et al., 2010). All the cDNAs have been verified by sequencing.

### 2.2 Cell culture and heterologous expression

The electrophysiological studies were performed in tsA-201 cells. The cells were cultured in Dulbecco's modified Eagle's medium (DMEM) supplemented with 10% fetal bovine serum, 1% Glutamax and 50 U/mL penicillin, 50 μg/mL streptomycin. The cells were transfected using FuGENE 6 in a ratio of 6:2 to DNA. The cDNAs of Ca_V_2.1, α_2_δ-1, β1b, the truncated domains and CD8 (transfection marker) were mixed in a ratio of 3:2:2:3:0.8. For the experiments where the effect of the N-terminus had been assessed, the DNA mixes contained Ca_V_2.1, α_2_δ-1, β1b, the truncated N-terminus constructs and CD8 in a ratio of 3:2:2:3:0.8. Finally to examine whether the N-terminus residues could restore the channel function, Ca_V_2.1 was co-transfected with α_2_δ-1, β1b, EA2, N-terminus constructs and CD8 (ratio 3:2:2:3:3:0.8).

For co-immunoprecipitation experiments, tsA-201 cells were transfected with Ca_V_2.2 HA, α_2_δ-1, β1b, Ca_V_2.2 Dom I–II and Ca_V_2.2-(43-95)-CAAX in a ratio of 3:2:2:3:3. In control experiments, Ca_V_3.1 Dom I–II was used in place of Ca_V_2.2 Dom I–II or GFP-CAAX was used in place of Ca_V_2.2-(43-95)-CAAX. When neither GFP-CAAX nor Ca_V_2.2-(43-95)-CAAX was included in the mix, the cDNA was maintained at 2 μg total by including empty pMT2 vector.

Neuro2A mouse neuroblastoma cells were used for the immunocytochemistry experiments. They were maintained in DMEM/Opti-MEM, 5% fetal bovine serum, 1% Glutamax, 50 U/mL penicillin and 50 μg/mL streptomycin. The cells were seeded on poly-l-lysine coated coverslips (0.25 mg/mL, Sigma-Aldrich) and were transfected with PolyJet, in a ratio of 3:1 to DNA. The DNA mixes consisted of pMT2 Ca_V_2.2 HA, pcDNA3 α_2_δ-1, pRK5 β1b and truncated domains in a ratio of 2:1:1:2.

### 2.3 DRG neuron preparation

The preparation of cultured dorsal root ganglion (DRG) neurons was performed as described previously (D'Arco et al., 2015). Briefly, DRG neurons isolated from P10 Sprague-Dawley rats were transfected by nucleofection following the manufacturer's instructions (Program G-13, Lonza). The cells were transfected with pcDNA3 empty vector and YFP for the control condition in a 4:0.8 ratio, or with pcDNA3-EA2, YFP and either pCDNA3 empty vector or pcDNA3 Ca_V_2.1-Nter-46-100-CAAX in a 2:2:0.8 ratio (2 μg of total DNA in all cases). DRGs were then plated on poly-l-lysine-coated coverslips (0.25 mg/mL, Sigma-Aldrich) and cultured in DMEM-F12 (Invitrogen) containing 10% FBS and 50 ng/mL NGF.

### 2.4 Electrophysiology

Whole-cell patch-clamp recordings were performed on tsA-201 cells 48 h after transfection and DRG neurons 4 days after transfection, at room temperature. The cells were replated prior to voltage-clamp experiments. Briefly, the neurons were incubated in a collagenase solution (0.2 mg/mL in serum free DMEM-F12) at 37 °C for 10 min. Neurons were then resuspended in DMEM-F12/FBS and plated on poly-l-lysine-coated coverslips. tsA-201 cells were dissociated using a non-enzymatic dissociation reagent (Sigma), resuspended in DMEM and plated on 35 mm dishes. Voltage-clamp experiments were performed 2–6 h after replating. For tsA-201 cells, patch pipettes were filled with a solution containing the following (in mM): 140 Cs-aspartate, 5 EGTA, 2 MgCl_2_, 0.1 CaCl_2_, 2 K_2_ATP and 10 HEPES, titrated to pH 7.2 with CsOH. The external solution contained the following (in mM): 150 tetraethylammonium bromide, 3 KCl, 1 NaHCO_3_, 1 MgCl_2_, 10 HEPES, 4 Glucose and 1 BaCl_2_, pH adjusted to 7.4 with Tris base. For the DRG recordings, the external solution contained the following (in mM): 150 tetraethylammonium bromide, 3 KCl, 1 NaHCO_3_, 1 MgCl_2_, 10 HEPES, 4 Glucose and 5 BaCl_2_, 1 μM Tetrodotoxin (TTX), 1 μM nifedipine and 1 μM ω-conotoxin GVIA to isolate the native P/Q type calcium currents. The currents were recorded using an Axopatch 200B amplifier (Axon instruments). Data were acquired with Clampex 8.2 and analysed with Clampfit 10.2 and Origin 7 software.

### 2.5 Immunocytochemistry

48 h after transfection, the Neuro-2a cells were washed twice with PBS and fixed with 4% paraformaldehyde (PFA) for 10 min. The cells were then incubated with rat *anti*-HA antibody (Roche) at a dilution of 1:500 in PBS with 1% Bovine Serum Albumin (BSA) for 1 h at room temperature. The cells were washed and incubated with a secondary *anti*-rat antibody conjugated to fluorescein isothiocyanate. Detection of the intracellular channel pool following surface labelling was performed after cell permeabilization with 0.2% Triton X-100 for 10 min. The coverslips were then washed with PBS and incubated with rabbit *anti*-Ca_v_2.2 antibody (polyclonal, affinity-purified antibody directed against the intracellular II–III linker) at a dilution of 1:500 for 1 h in PBS with 1% BSA. The cells were then incubated with secondary antibody, *anti*-Rabbit Alexa 594 (Abcam) at a dilution of 1:500.

Four days after transfection, the DRG neurons were washed and fixed with 4% PFA for 10 min. The neurons were then permeabilized with 0.2% Triton X-100 for 10 min. The cells were incubated in PBS blocking solution containing 10% goat serum (GS) and 5% BSA, for 30 min. The primary *anti*-Ca_V_2.1 antibody (Alomone Labs) in PBS, containing 5% GS and 2.5% BSA, was applied at 4 °C overnight. The cells were washed and the secondary antibody *anti*-rabbit Alexa 594 (Abcam) was applied in PBS containing 5% GS and 2.5% BSA, for 1 h at room temperature. For all cells, the coverslips were washed and mounted on slides using Vectashield (Vector Laboratories) to reduce photobleaching. Imaging was performed using a confocal laser-scanning microscope (Zeiss).

Cell surface expression was measured using ImageJ, by manually tracing the surface of the cells represented by the HA signal. The background signal was measured in an area of the image lacking cells and subtracted from the measurements.

### 2.6 Immunoprecipitation and western-blotting

Cells were lysed using 1% Igepal in PBS in the presence of 25 mM *N*-ethylmaleimide (NEM) and protease inhibitors for 30 min on ice, and whole cell lysates (WCL) were collected after centrifugation (14,000 ×* g*, for 30 min at 4 °C). The total protein concentration was determined (Bradford assay, Bio-Rad) for each sample. For co-immunoprecipitation, WCL (containing 1 mg protein) was pre-cleared on 30 μL protein A/G Plus Agarose (Santa Cruz) for 2 h at 4 °C. Agarose beads were discarded and supernatants were incubated with rat *anti*-HA (Roche) at a dilution of 1:100 overnight at 4 °C. Immunoprecipitated proteins were captured on 50 μL of protein A/G beads for 2 h at 4 °C. The beads were washed three times with PBS containing 0.1% Igepal. The beads, as well as the WCL samples, were incubated for 15 min at 55 °C with 100 mM DTT, 25 mM NEM and 2 × Laemmli sample buffer. Eluted co-immunoprecipitated proteins and WCL samples were resolved by SDS–PAGE 3–8% Tris-Acetate gels and then transferred to polyvinylidene fluoride membranes. Membranes were incubated in blocking buffer (10 mM Tris pH 7.4, 500 mM NaCl, 0.5% Igepal and 3% BSA) for 1 h, followed by incubation with rabbit *anti*-Ca_V_2.2 (against the II–III loop) overnight at a dilution of 1:1000 at 4 °C. The secondary antibody, goat *anti*-rabbit coupled to horseradish peroxidise, was incubated at a dilution of 1:2000 for 1 h at room temperature. Proteins were detected using the enhanced ECL Plus reagent (GE Healthcare) on a Typhoon 9410 scanner (GE Healthcare).

### 2.7 Statistical analysis

Statistical analysis between groups was performed using one-way ANOVA with Bonferroni post-hoc test, using Graphpad Prism software. Statistical analysis between two groups (Fig. 4) was carried out using Student's *t* test.

## 3 Results

### 3.1 Lack of cell surface expression of truncated channels

**Fig. 1:**
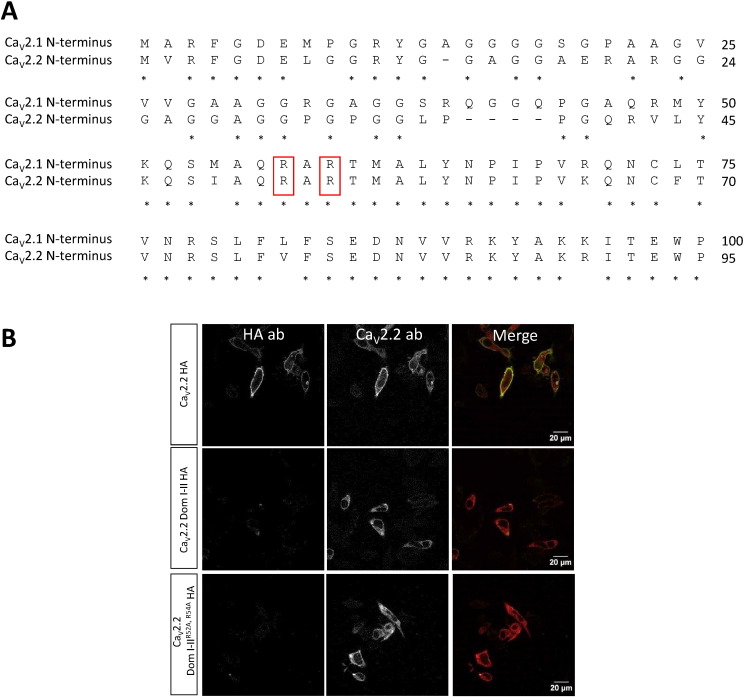
Comparison of Ca_V_2.1 and Ca_V_2.2 N-termini sequences and expression of truncated domains. (A) The sequences were aligned with DNASTAR program using Clustal V. Gaps are shown as (–), identical amino acids are indicated as ‘*’. The conserved arginine residues are indicated by red rectangles. (B) Representative confocal images of Neuro2A cells expressing Ca_V_2.2 HA (top), Ca_V_2.2 Dom I–II HA (middle) and Ca_V_2.2 Dom I–II^R52A, R54A^ HA (bottom). Surface staining was obtained by using HA antibody before cell permeabilization (left panel). The intracellular Ca_V_2.2 constructs were detected in permeabilized cells using a Ca_V_2.2 antibody that targets the II–III loop (middle panel). The merged images are shown in the right panel. Scale bar; 20 μm.

The Ca_V_2.1 and Ca_V_2.2 N-termini show a high degree of homology (Fig. 1A). Here we assessed the role of the two conserved arginine residues (indicated by the red rectangles in Fig. 1A) in the dominant-negative suppression of Ca_V_2 channels by truncated two domain constructs. We initially monitored the surface expression of the full-length and truncated Ca_V_2.2 harbouring an exofacial HA tag (Cassidy et al., 2014), co-expressed with the auxiliary subunits α_2_δ-1 and β1b. Ca_V_2.2 was used to monitor cell surface expression, since in our hands Ca_V_2.1 with an exofacial tag (Watschinger et al., 2008) was not well exposed on the cell surface. While the full-length Ca_V_2.2 channel was found to be expressed on the cell surface of non-permeabilized Neuro2A cells, as shown by the HA signal (Fig. 1B), a truncated Ca_V_2.2 construct, consisting of Dom I–II and the II–III loop was retained intracellularly, and no surface HA signal was detected (Fig. 1B). Ca_V_2.2 Dom I–II^R52A, R54A^, in which the key arginine residues identified in Fig. 1A were substituted by alanine, was also not trafficked to the surface (Fig 1B). The fact that the truncated two domain Ca_V_2.2 constructs are not expressed at the cell surface is not surprising as it has already been shown that the truncated mutant Ca_V_2.1 channels alone do not form functional channels (Jouvenceau et al., 2001). However, for Ca_V_2.2 and Ca_V_1.2, co-expression of complementary truncated domain pairs was found to give rise to currents, although their amplitude was smaller than the full-length channel current (Raghib et al., 2001, Michailidis et al., 2014), indicating that channel fragments can interact and fold appropriately, when all the domains are present.

### 3.2 Role of the N-terminal arginine motif in the suppressive effect of truncated channels

**Fig. 2:**
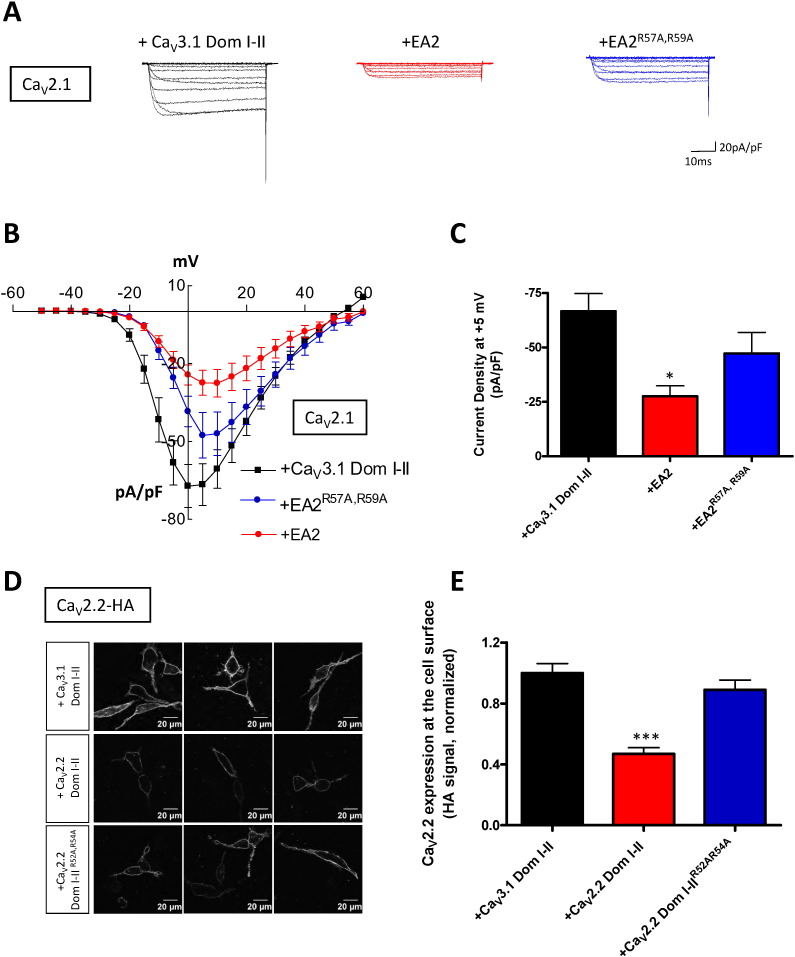
Effect of truncated domains on Ca_V_2 channel expression. (A–C) Effect of the truncated domains on Ca_V_2.1 currents. tsA-201 cells expressed full-length Ca_V_2.1 with Ca_V_3.1 Dom I–II as a control (black, control), EA2 (red) or EA2^R57A, R59A^ (blue), together with α_2_δ-1 and β1b subunits. (A) Representative traces for the three conditions were evoked by 50 ms step depolarizations between − 50 and + 60 mV from a holding potential of − 80 mV. The currents are normalised to the cell capacitance. (B) Mean current-voltage relationships for Ca_V_2.1 with Ca_V_3.1 Dom I–II (black squares, control), Ca_V_2.1 with EA2 (red circles) or Ca_V_2.1 with EA2^R57A, R59A^ (blue circles). (C) Bar chart of mean current densities (pA/pF) at + 5 mV ± SEM for Ca_V_2.1 with Ca_V_3.1 Dom I–II (black, control, n = 20), Ca_V_2.1 with EA2 (red, n = 24) and Ca_V_2.1 with EA2^R57A, R59A^ (blue, n = 21). Statistical analysis *p < 0.05 vs control. (D–E) Effect of the truncated constructs on Ca_V_2.2 HA cell surface expression. Neuro2A cells expressed full-length Ca_V_2.2 with Ca_V_3.1 Dom I–II, Ca_V_2.2 Dom I–II or Ca_V_2.2 Dom I–II^R52A, R54A^, together with α_2_δ-1 and β1b subunits. (D) Three examples of confocal images of Neuro2A cells expressing Ca_V_2.2 HA with Ca_V_3.1 Dom I–II (top), Ca_V_2.2 HA with Ca_V_2.2 Dom I–II (middle) or Ca_V_2.2 HA and Ca_V_2.2 Dom I–II^R52A, R54A^ (bottom). Surface staining was obtained by using HA antibody in non-permeabilized cells. (E) Cell surface expression of Ca_V_2.2 was quantified based on the HA signal. Bar chart of mean of cell surface expression ± SEM for Ca_V_2.2 HA with Ca_V_3.1 Dom I–II (black, control), Ca_V_2.2 HA with Ca_V_2.2 Dom I–II (red) or Ca_V_2.2 HA with Ca_V_2.2 Dom I–II^R52A, R54A^ (blue). The data were pooled from 4 independent experiments; more than 100 cells were included in the analysis for each condition. Statistical analysis: ***p < 0.001 vs control.

We next examined the suppressive effect of the truncated constructs on Ca_V_2.1 and Ca_V_2.2 channel function and trafficking. We first investigated the effect of the substitution of R57A, R59A in an EA2 mutant (Ca_V_2.1-P1217fs), containing only the first two domains and the intracellular II–III loop; this mutant will be referred as EA2 throughout the paper. In all our studies, the equivalent first two domains of Ca_V_3.1 were used as a negative control as this was shown previously not to cause dominant-negative inhibition of Ca_V_2.2 channels (Page et al., 2010). This was also found in the present study for Ca_V_2.1 as shown by the unchanged current densities at + 5 mV in the presence of either Ca_V_3.1 Dom I–II (− 66.6 ± 8.0 pA/pF) or GFP-CAAX (− 75.3 ± 13.0 pA/pF) (data from Fig. 2C and Fig. 5C).

As shown in Fig. 2A–C, while the EA2 mutant exerted a strong dominant-negative effect, significantly reducing Ca_V_2.1 current density at + 5 mV by 63% (control: − 66.6 ± 8.0 pA/pF; EA2: − 27.4 ± 4.8 pA/pF), the EA2^R57A, R59A^ construct (− 47.2 ± 9.6 pA/pF) produced a non-significant dominant-negative inhibition (Fig. 2C). There was no difference in the voltage for 50% activation (V_50_) (data not shown). This differential effect was confirmed for cell surface expression of full-length Ca_V_2.2, which was significantly reduced by co-expression with Ca_V_2.2 Dom I–II but not with Ca_V_2.2 Dom I–II^R52A, R54A^ (Fig. 2D, E). This confirms that the N-terminal RAR motif in Ca_V_2.1 and Ca_V_2.2 is involved in the process underlying the dominant-negative suppression by the truncated channels.

**Fig. 3:**
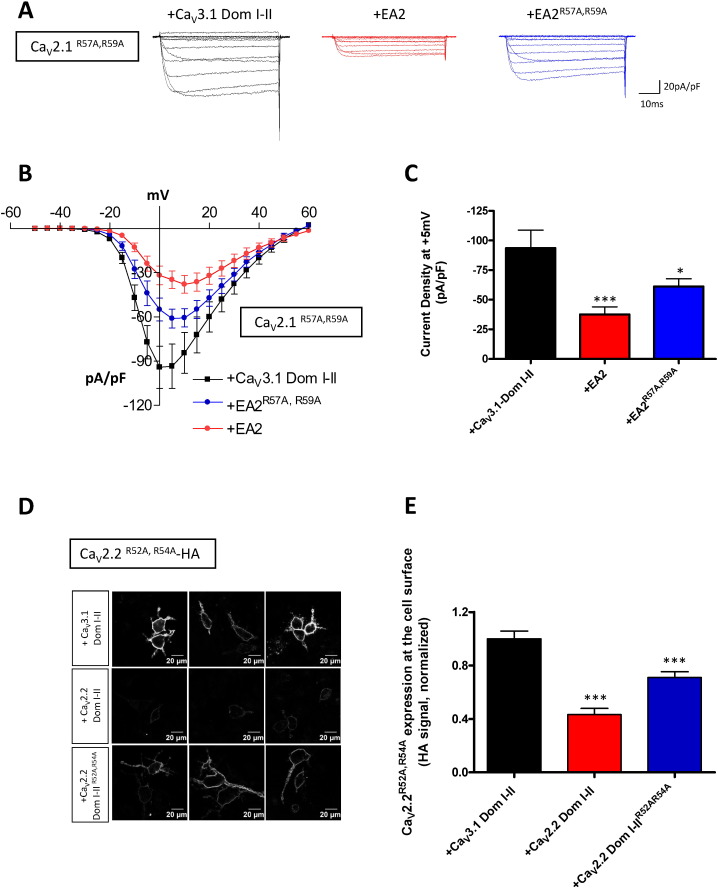
Effect of truncated domains on N-terminally mutated Ca_V_2 channel expression. (A-C) Effect of the truncated constructs on Ca_V_2.1^R57A, R59A^ currents. (A) tsA-201 cells were co-transfected with full-length Ca_V_2.1^R57A, R59A^/α_2_δ-1/β1b and Ca_V_3.1 Dom I–II (control, black), EA2 (red) or EA2^R57A, R59^ (blue). Representative traces were evoked by 50 ms step depolarizations between − 50 and + 60 mV from a holding potential of − 80 mV. The currents are normalised to the cell capacitance. (B) Mean current-voltage relationships for Ca_V_2.1^R57A, R59A^ and Ca_V_3.1 Dom I–II (black squares, control), Ca_V_2.1^R57A, R59A^ and EA2 (red circles) or Ca_V_2.1^R57A, R59A^ and EA2^R57A, R59A^ (blue circles). (C) Mean current densities (pA/pF) at + 5 mV ± SEM for Ca_V_2.1^R57A, R59A^ with Ca_V_3.1 Dom I–II (black, control, n = 10), Ca_V_2.1^R57A, R59A^ with EA2 (red, n = 18) or Ca_V_2.1^R57A, R59A^ with EA2^R57A, R59A^ (blue, n = 13). Statistical analysis: *p < 0.05, ***p < 0.001 vs control. (D–E) Effect of the truncated constructs on Ca_V_2.2^R52A, R54A^ HA cell surface expression. Neuro2A cells expressed Ca_V_2.2^R52A, R54A^ HA/α_2_δ-1/β1b with Ca_V_3.1 Dom I–II (control), Ca_V_2.2 Dom I–II or Ca_V_2.2 Dom I–II^R52A, R54A^. (D) Three examples of confocal images Ca_V_2.2^R52A, R54A^ HA with Ca_V_3.1 Dom I–II (top), Ca_V_2.2^R52A, R54A^ HA with Ca_V_2.2 Dom I–II (middle) or Ca_V_2.2^R52A, R54A^ HA with Ca_V_2.2 Dom I–II^R52A, R54A^ (bottom). (E) Cell surface expression was quantified based on the HA signal. The data represent mean ± SEM for Ca_V_2.2^R52A, R54A^ HA with Ca_V_3.1 Dom I–II (black, control), Ca_V_2.2^R52A, R54A^ HA with Ca_V_2.2 Dom I–II (red) or Ca_V_2.2^R52A, R54A^ HA with Ca_V_2.2 Dom I–II^R52A, R54A^ (blue). The data were pooled from 4 independent experiments; more than 100 cells were included in the analysis for each condition. Statistical analysis: ***p < 0.001 vs control.

We next used full-length channel constructs carrying the arginine-to-alanine substitutions (Ca_V_2.1^R57A, R59A^ and Ca_V_2.2^R52A, R54A^), and assessed the dominant-negative effect of the truncated constructs. As shown in Fig. 3A–C, co-expression of the EA2 mutant significantly reduced Ca_V_2.1^R57A, R59A^ current by 60% (control: − 93.5 ± 15.0 pA/pF; EA2: − 37.5 ± 6.3 pA/pF), indicating that the full-length channel still interacts with the EA2 mutant despite the fact that its own arginine motif was disrupted. However there was still a dominant-negative effect when Ca_V_2.1^R57A, R59A^ was co-expressed with EA2^R57A, R59A^ (− 61.1 ± 6.4 pA/pF), albeit reduced compared to the EA2 condition (Fig. 3B). It is of interest that Ca_V_2.1^R57A, R59A^ exhibited larger currents than wild-type Ca_V_2.1 (Fig. 3C, compared to Fig. 2C), suggesting that there may be a tonic inhibitory effect that is relieved by mutating the N-terminus.

Similar findings were obtained for cell surface expression of Ca_V_2.2. The cell surface expression of full-length Ca_V_2.2^R52A, R54A^ was significantly reduced by Ca_V_2.2 Dom I–II, and also by Ca_V_2.2 Dom I–II^R52A, R54A^ (Fig. 3D, E).

Thus the RAR motif in the N-terminus of the truncated two domain constructs, but not the full-length channels, is directly involved in the suppressive effect of the truncated constructs. Because this inhibition is mediated by the interaction between the full-length and truncated constructs, we hypothesised that the N-terminus of the truncated construct is essential for the truncated channels to interact with the full-length channels. We therefore tested whether the co-expression of the key N-terminal peptide would prevent this deleterious interaction.

### 3.3 Co-expression of an N-terminus construct disrupted the interaction of the full-length Ca_V_2.2 with the truncated domains

In this study we used an N-terminal construct with a CAAX myristoylation motif attached to its C-terminus to allow its association with intracellular lipid bilayers (Page et al., 2010). An N-terminally truncated N-terminus construct still containing the RAR motif was used, as it has been shown that the full-length N-terminus itself significantly reduced calcium currents (Page et al., 2010). In Ca_V_2.2-(43-95)-CAAX, the first 42 residues containing the glycine-rich region were removed (Fig. 1A).

**Fig. 4:**
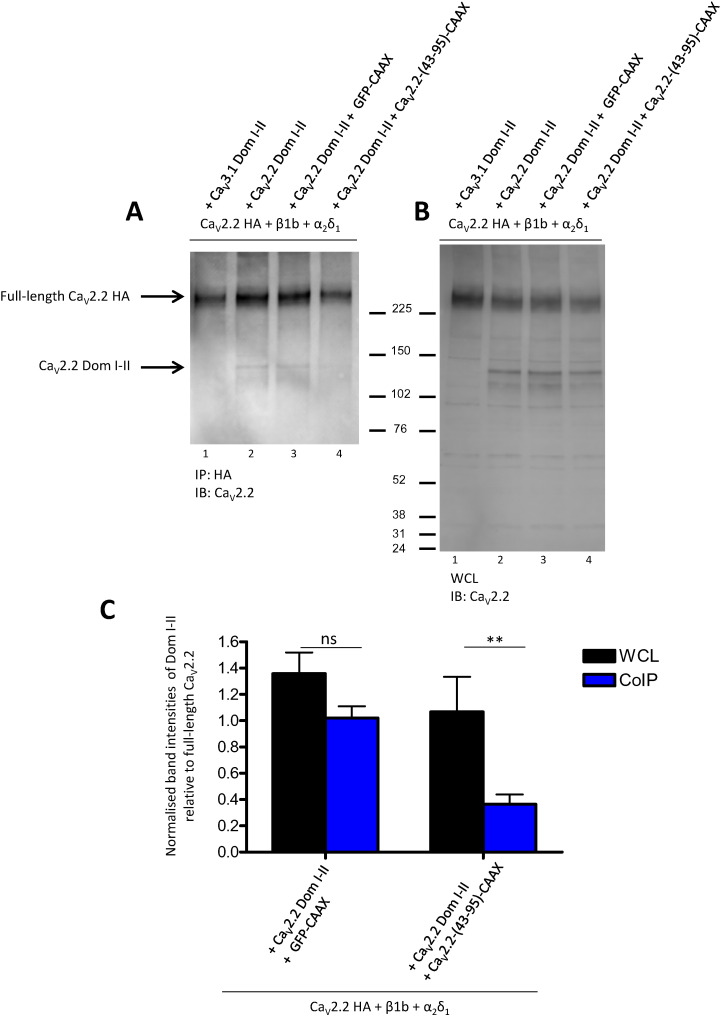
Co-immunoprecipitation of full-length and truncated Ca_V_2.2 is disrupted by the N-terminal construct. A: Representative Western blot showing proteins immunoprecipitated with rat *anti*-HA antibody and immunoblotted with *anti*-Ca_V_2.2. tsA-201 cells expressed Ca_V_2.2 HA, α2δ-1 and β1b, together with Ca_V_3.1 Dom I–II (lane 1), Ca_V_2.2 Dom I–II (lane 2), Ca_V_2.2 Dom I–II plus GFP-CAAX (lane 3) or Ca_V_2.2 Dom I–II plus Ca_V_2.2-(43-95)-CAAX (lane 4). This result is representative of 3 independent experiments. B: Western-blot showing 30 μg WCL of the same samples shown in A. C: Quantification of Western blots. Band intensities for Ca_V_2.2 Dom I–II were measured using ImageJ and normalised for expression of full-length Ca_V_2.2 HA for each condition and relative to the condition of Ca_V_2.2 HA + Ca_V_2.2 Dom I–II (lane 2 in A and B). Data are mean band intensities (+ SEM) from three co-immunoprecipitation experiments from three different transfections. Statistical analysis: ns = non-significant difference, **p < 0.01.

We performed co-immunoprecipitation experiments to determine if Ca_V_2.2-(43-95)-CAAX interferes with the association of full-length Ca_V_2.2 HA with Ca_V_2.2 Dom I–II. WCL were immunoprecipitated with HA antibody and then probed with a Ca_V_2.2 antibody that targets the II–III loop, present in both the full-length and the truncated channel (Fig. 4A). As shown in Fig. 4, full-length Ca_V_2.2 HA was able to co-immunoprecipitate Ca_V_2.2 Dom I–II (Fig. 4A, band indicated in lane 2). Ca_V_2.2-(43-95)-CAAX (lane 4), but not GFP-CAAX used as a control (lane 3), interfered with the interaction between full-length Ca_V_2.2 HA and Ca_V_2.2 Dom I–II, as indicated by the absence of the band corresponding to Ca_V_2.2 Dom I–II in lane 4 (Fig. 4A). Three independent experiments were performed and the bands corresponding to Ca_V_2.2 Dom I–II were quantified and normalised to full-length Ca_V_2.2 expression for each condition, in both co-immunoprecipitation and WCL western-blots (Fig. 4A, B). The mean data (Fig. 4C) show the normalised values with respect to the Ca_V_2.2 HA + Ca_V_2.2 Dom I–II condition (lane 2 in Fig. 4A and B). The results show that Dom I–II co-immunoprecipitation with Ca_V_2.2 was significantly decreased compared to the WCL in the presence of Ca_V_2.2-(43-95)-CAAX, indicating that the N-terminus disrupted the association of full-length Ca_V_2.2 and Ca_V_2.2 Dom I–II. In addition, the presence of the N-terminus construct did not affect the total amount of Ca_V_2.2 full-length channel, indicated by the unchanged amount of full-length channel band in lane 3 and 4 (Fig. 4B).

We next examined whether uncoupling the deleterious interaction and thus releasing the full-length channel from its association with the truncated protein by expressing the key N-terminus residues can restore the channel's function and trafficking.

### 3.4 Co-expression of the free N-terminus did not alter the function of Ca_V_2.1

**Fig. 5:**
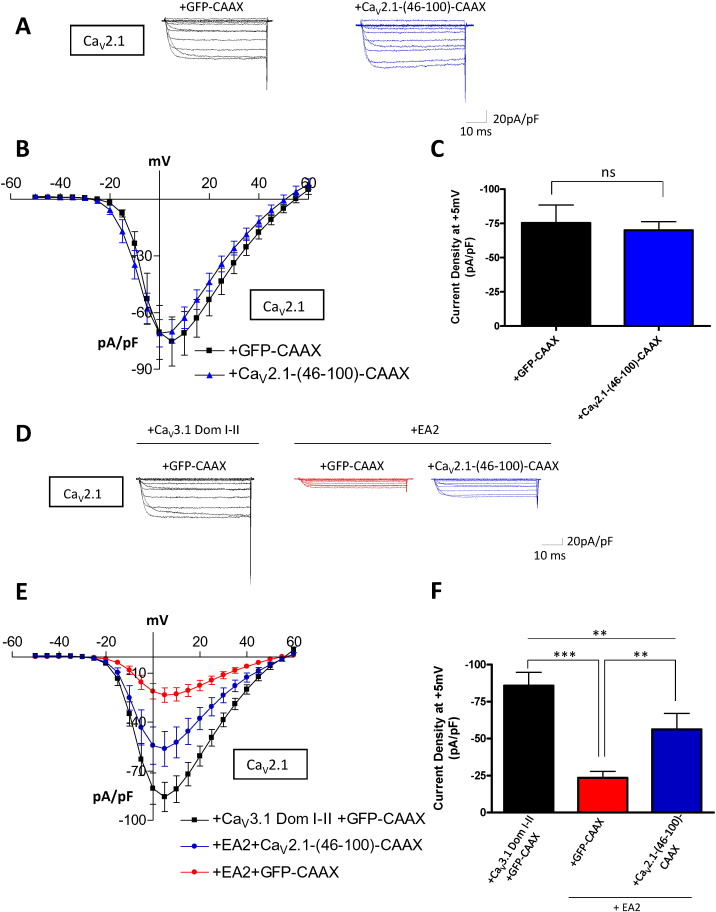
Effect of the N-terminus on Ca_V_2.1 current and suppression by EA2. (A–C) Effect of the N-terminal constructs on Ca_V_2.1/β1b/α_2_δ-1 currents. (A) Representative traces were evoked by 50 ms step depolarizations between − 50 and + 60 mV from a holding potential of − 80 mV for Ca_V_2.1/α_2_δ-1/β1b currents, expressed together with GFP-CAAX (black, control), or Ca_V_2.1-(46-100)-CAAX (blue). (B) Mean current-voltage relationships of Ca_V_2.1 and GFP-CAAX (black squares, control, n = 13) or Ca_V_2.1 and Ca_V_2.1-(46-100)-CAAX (blue triangles, n = 12). (C) Mean current density at + 5 mV ± SEM. Statistical analysis: ns = non-significant difference. (D–E) Effect of the N-terminal constructs on the suppressive effect of EA2 on Ca_V_2.1 currents. (D) Representative traces from tsA-201 cells transfected with Ca_V_2.1/β1b/α_2_δ-1, together with Ca_V_3.1 Dom I–II and GFP-CAAX (black, control), Ca_V_2.1, EA2 and GFP-CAAX (red) or Ca_V_2.1, EA2 and Ca_V_2.1-(46-100)-CAAX (blue). (E) Mean current-voltage relationships for Ca_V_2.1, Ca_V_3.1 Dom I–II and GFP-CAAX (black squares, control, n = 25), Ca_V_2.1 and EA2 plus GFP-CAAX (red circles, n = 24) or Ca_V_2.1 and EA2 plus Ca_V_2.1-(46-100)-CAAX (blue circles, n = 15). (F) Mean current density at + 5 mV ± SEM. Statistical analysis: **p < 0.01, ***p < 0.001.

First, we evaluated whether there was any direct effect of the N-terminal constructs on channel function. When co-transfected with the full-length channel, the truncated Ca_V_2.1 N-terminus Ca_V_2.1-(46-100)-CAAX corresponding to Ca_V_2.2-(43-95)-CAAX did not affect Ca_V_2.1 function, as shown by the unchanged current densities compared to control cells co-transfected with the control protein GFP-CAAX (control: − 75.3 ± 13.0 pA/pF; Ca_V_2.1-(46-100)-CAAX: − 69.0 ± 6.1 pA/pF; Fig. 5A–C). This indicates that the N-terminal fragment, lacking the initial residues 1–45, does not alone interact with the full-length channel sufficiently to cause inhibition.

### 3.5 Co-expression of the N-terminal construct partially restored the function of Ca_V_2.1

We next assessed whether the expression of the Ca_V_2.1 N-terminal construct, Ca_V_2.1-(46-100)-CAAX, might restore Ca_V_2.1 function by interfering with the dominant-negative effect of EA2.

As shown in Fig. 5D–F, co-expression of Ca_V_2.1-(46-100)-CAAX partially reversed the suppression of Ca_V_2.1 current by EA2. Indeed, the current density was increased 2.4-fold for the condition in which EA2 was co-expressed with Ca_V_2.1-(46-100)-CAAX (control: − 85.7 ± 9.0 pA/pF; Ca_V_2.1-(46-100)-CAAX: − 56.2 ± 10.7 pA/pF), compared to the condition in which EA2 was co-expressed with GFP-CAAX (23.3 ± 4.5 pA/pF; Fig. 5F). It is likely that Ca_V_2.1-(46-100) had this effect by disrupting or preventing the interaction between the full-length channel and the EA2 construct.

### 3.6 Co-expression of the N-terminus construct restored the expression of Ca_V_2.2 at the cell surface

**Fig. 6:**
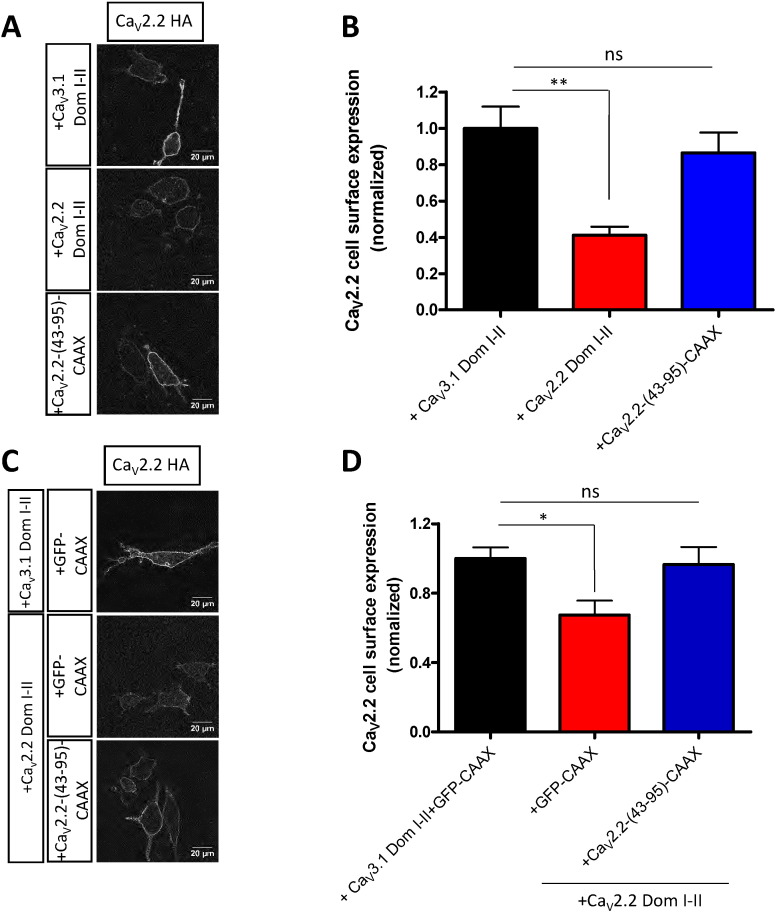
Effect of the Ca_V_2.2 N-terminus and truncated domains on Ca_V_2.2 HA trafficking. (A) Representative confocal images of Neuro2A cells transfected with Ca_V_2.2 HA/α_2_δ-1/β1b in the presence of Ca_V_3.1 Dom I–II (top panel), Ca_V_2.2-Dom I–II (middle panel) or Ca_V_2.2-(43-95)-CAAX (bottom panel). Surface staining was obtained using HA antibody in non-permeabilized cells. (B) Ca_V_2.2 cell surface expression of was quantified based on the HA signal. Mean cell surface expression ± SEM for Ca_V_2.2 HA and Ca_V_3.1 Dom I–II (black, control), Ca_V_2.2 HA and Ca_V_2.2-Dom I–II (red) or Ca_V_2.2 HA and Ca_V_2.2-(43-95)-CAAX (blue). The data are pooled from 3 independent experiments; more than 50 cells have been included in the analysis for each condition. Statistical analysis: **p < 0.01, ns = non-significant difference. (C) Representative confocal images of Neuro2A cells, showing cell surface expression of Ca_V_2.2 HA in the presence of Ca_V_3.1 Dom I–II and GFP-CAAX (top panel), Ca_V_2.2 HA, Ca_V_2.2-Dom I–II and GFP-CAAX (middle panel) or Ca_V_2.2 HA, Ca_V_2.2-Dom I–II and Ca_V_2.2-(43-95)-CAAX (bottom panel). Surface staining was obtained by using HA antibody before cell permeabilization. (D) Cell surface expression of Ca_V_2.2 was quantified based on the HA signal using ImageJ software. Bar chart of mean ± SEM cell surface expression of Ca_V_2.2 HA and Ca_V_3.1 Dom I–II and GFP-CAAX (black), Ca_V_2.2 HA, Ca_V_2.2 Dom I–II and GFP-CAAX (red) or Ca_V_2.2 HA, Ca_V_2.2 Dom I–II and Ca_V_2.2-(43-95)-CAAX (blue). The data are pooled from 3 independent experiments and more than 50 cells have been included in the analysis for each condition. Statistical analysis: *p < 0.05, ns = non-significant difference.

We next wanted to examine whether the suppression of Ca_V_2.2 trafficking by Ca_V_2.2 Dom I–II is also disrupted by co-expression of the equivalent Ca_V_2.2 N-terminal peptide. In parallel with the result found for Ca_V_2.1 currents, we found firstly that Ca_V_2.2-(43-95)-CAAX, unlike Ca_V_2.2 Dom I–II, did not alter the cell-surface expression of Ca_V_2.2 when they were co-expressed, as shown by the unchanged mean intensity of the HA signal at the cell surface (Fig. 6A, B). Furthermore, co-expression of Ca_V_2.2-(43-95)-CAAX restored Ca_V_2.2 cell surface expression by decreasing the deleterious effect of Ca_V_2.2 Dom I–II (Fig. 6C, D). These results nicely recapitulate the Ca_V_2.1 results shown in Fig. 5.

### 3.7 Co-expression of the N-terminal construct did not restore the function of Ca_V_2.1^R57A, R59A^

**Fig. 7:**
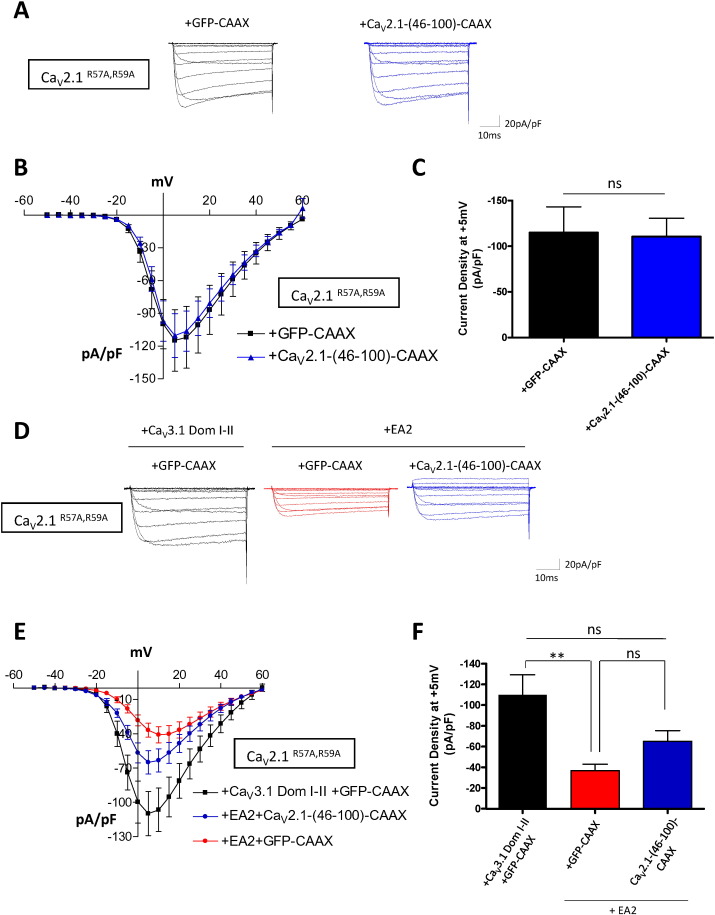
Effect of the N-terminal construct on Ca_V_2.1^R57A, R59A^ current and suppression by EA2. (A) Representative traces of the currents evoked by Ca_V_2.1^R57A, R59A^ in presence of GFP-CAAX (black) or Ca_V_2.1-(46-100)-CAAX (blue). (B) Mean current-voltage relationships of Ca_V_2.1 and GFP-CAAX (black squares, n = 11) or Ca_V_2.1 and Ca_V_2.1-(46-100)-CAAX (blue triangles, n = 12). (C) Mean current density at + 5 mV for the two conditions. Statistical analysis: ns = non-significant difference. (D–F) Effect of the N-terminal construct on the suppressive effect of EA2 on Ca_V_2.1^R57A, R59A^ current. (D) Representative traces of tsA-201 cells transfected with Ca_V_2.1^R57A, R59A^, Ca_V_3.1 Dom I–II and GFP-CAAX for the control condition (black) or Ca_V_2.1 and EA-2 in the presence of GFP-CAAX (red), or Ca_V_2.1-(46-100)-CAAX (blue). (E) Mean current-voltage relationships of Ca_V_2.1, Ca_V_3.1 Dom I–II and GFP-CAAX (black squares, n = 14), Ca_V_2.1, EA2 and GFP-CAAX (red circles, n = 26) or Ca_V_2.1, EA2 and Ca_V_2.1-(46-100)-CAAX (blue circles, n = 21). (F) Mean current density at + 5 mV ± SEM for Ca_V_2.1, Ca_V_3.1 Dom I–II and GFP-CAAX (black, n = 10), Ca_V_2.1, EA2 and GFP-CAAX (red, n = 27) or Ca_V_2.1, EA2 and Ca_V_2.1-(46-100)-CAAX (blue, n = 23). Statistical analysis: **p < 0.01, ns = non-significant difference.

Since we found in this study that the EA2 mutant still exerted a dominant-negative effect on Ca_V_2.1^R57A, R59A^ (Fig. 3A–C), we next examined whether it was possible to perturb this suppressive effect by co-expressing Ca_V_2.1-(46-100)-CAAX. We first showed that this construct did not have a direct effect on Ca_V_2.1^R57A, R59A^ currents (control: 114.0 ± 28.0 pA/pF; Ca_V_2.1-(46-100)-CAAX: 110.0 ± 20.0 pA/pF; Fig. 7A–C). Furthermore, the dominant-negative effect of EA2 on Ca_V_2.1^R57A, R59A^ currents was not significantly reversed by Ca_V_2.1-(46-100)-CAAX (control: 109.9 ± 19.2 pA/pF; EA2 + GFP-CAAX: − 36.8 ± 6.0; EA2 + Ca_V_2.1-(46-100)-CAAX: − 65.0 ± 8.8 pA/pF; Fig.7D–F). This likely indicates that the suppressive effect of EA2 on Ca_V_2.1^R57A, R59A^ is more robust, and less easily disrupted.

### 3.8 Co-expression of Ca_V_2.1 N-terminus restored P/Q current suppressed by EA2 in DRG neurons

**Fig. 8:**
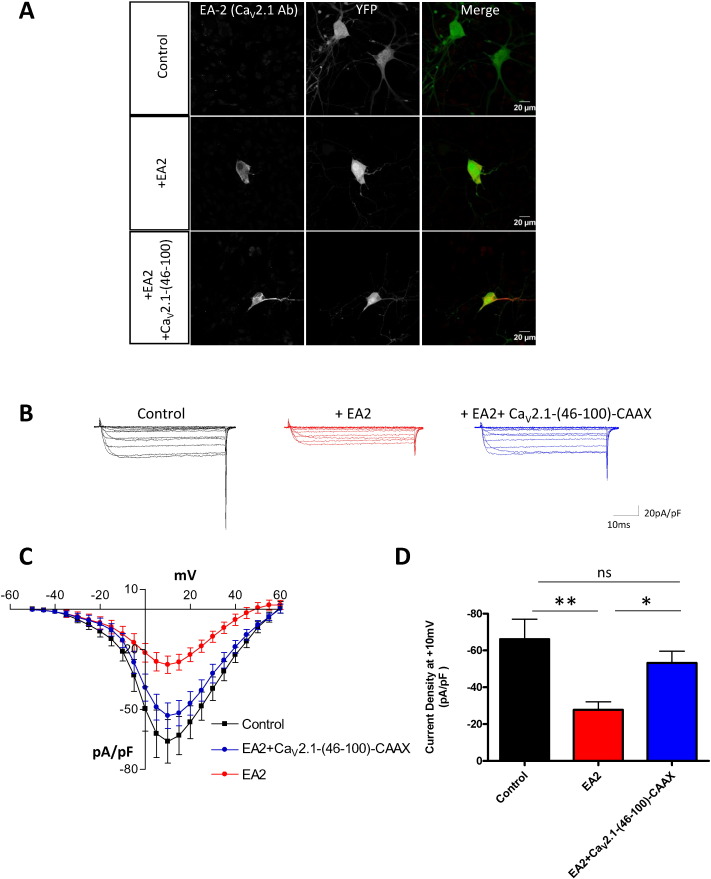
Rescue by the free N-terminus of the suppression by EA2 of endogenous P/Q type current in DRG neurons. (A) Confocal images of DRG neurons transfected with empty pcDNA3 vector and YFP (control, top panel), EA2, pcDNA3 empty vector and YFP (middle panel) or EA2, Ca_V_2.1-(46-100)-CAAX and YFP (bottom panel). The neurons were permeabilized and stained with Ca_V_2.1 antibody that targets the II–III loop. (B) Representative traces of endogenous P/Q current. The DRG neurons were co-transfected with empty pcDNA3 vector and YFP (black); EA2 and pcDNA3 empty vector and YFP (red) or EA2, Ca_V_2.1-(46-100)-CAAX and YFP (blue). The P/Q-type current was isolated pharmacologically. Currents were evoked by 50 ms step depolarizations between − 50 and + 60 mV from a holding potential of − 80 mV. The charge carrier was 5 mM Ba^2 +^. (C) Current-voltage relationships of DRG neurons transfected with pcDNA3 empty vector and YFP (black squares, n = 13), EA2, pcDNA3 empty vector and YFP (red circles, n = 10) or EA2, Ca_V_2.1-(46-100)-CAAX and YFP (blue circles, n = 15). (D) Current density at + 10 mV ± SEM for pcDNA3 empty vector and YFP (black, n = 13), EA2, pcDNA3 empty vector and YFP (red, n = 10) or EA2, Ca_V_2.1-(46-100)-CAAX and YFP (blue, n = 15). Statistical analysis: *p < 0.05, **p < 0.01, ns = non-significant difference.

We then examined whether Ca_V_2.1-(46-100)-CAAX could restore the endogenous P/Q-type current in DRG neurons co-transfected with the EA2 mutant. Firstly, DRG neurons were transfected with YFP as a transfection marker and empty vector, EA2 or EA2 plus Ca_V_2.1-(46-100)-CAAX, and stained with a Ca_V_2.1 antibody that targets the II–III loop present in EA2, to demonstrate its expression (Fig. 8A). The native Ca_V_2.1 could not be detected, probably because of its low level of expression. We performed experiments 4 days after transfection in the presence of 1 μM nifedipine and 1 μM ω-conotoxin GVIA to block L- and N-type channels, respectively, in order to isolate native P/Q-type calcium currents. The electrophysiological data showed that the expression of EA2 mutant induced, as expected, a reduction of the native P/Q-type current greater than 50% (control: − 66.0 ± 10.9 pA/pF; EA2: − 27.6 ± 4.3 pA/pF; Fig. 8B–D). Importantly, this reduction was almost completely prevented when Ca_V_2.1-(46-100)-CAAX was co-expressed (− 65.0 ± 8.8 pA/pF; Fig. 8B–D). This finding further reinforces the view that key N-terminal residues interfere with the dominant-negative effect of EA2.

## 4 Discussion

In this study we show for the first time that a key N-terminal peptide is able to interfere with the suppressive effect of the two domain truncated Ca_V_2 constructs mimicking EA2 mutant channels, and hence partially restore the functional expression of P/Q-type channels. This may pave the way for development of future treatments to prevent or disrupt the deleterious effect of the truncated channels.

Mutations in the *CACNA1A* gene encoding the pore-forming subunit of the Ca_V_2.1 channel cause several neurological disorders, in particular FMH1, SCA6 and EA2 (Pietrobon, 2010, Jen, 2008). EA2 is a unique channelopathy, as it is frequently associated with mutations leading to truncation of the protein. Although EA2 is episodic in nature, it may also be associated with progressive symptoms, and there are limited treatment options available (Jen, 2008). EA2 is a dominant disorder, and although haploinsufficiency was originally thought to be its cause, there is increasing evidence for a dominant-negative mechanism (Jouvenceau et al., 2001, Page et al., 2004, Cao et al., 2004, Mezghrani et al., 2008). *Cacna1a* knockout mice, and the naturally occurring *cacna1a* mutant mouse *tottering*, have been widely studied and show absence epilepsy, ataxia and paroxysmal dyskinesia, with progressive loss of cerebellar Purkinje cells (Fletcher et al., 1996, Fletcher et al., 2001, Shirley et al., 2008). However the heterozygous knockout and mutant mice show no evidence of neurodegeneration, and thus the null mutation is recessive (Fletcher et al., 2001), except in the case of the semi-dominant tottering^5J^ (Miki et al., 2008). Furthermore postnatal knockout of Ca_V_2.1 in Purkinje cells produces similar effects to the full knockout, in terms of neurological deficit (Mark et al., 2011). It is also of interest that siRNA knock-down of P/Q-type channels in adult mouse cerebellum resulted in mild impairment that could be enhanced by activation of β-adrenoceptors, mimicking stress (Salvi et al., 2014).

Our study confirms that an arginine-alanine-arginine (RAR) motif in the N-terminus is involved in the suppressive effect of the truncated channels. Interestingly, we found that disrupting the arginine motif in the full-length Ca_V_2.1^R57A, R59A^ and Ca_V_2.2^R52A, R54A^ channels did not impede channel function or expression at the cell surface, respectively. It is also worth mentioning that Ca_V_2.1^R57A, R59A^ evoked a larger current compared to wild-type Ca_V_2.1. The level of expression of Ca_V_2.1^R57A, R59A^ and Ca_V_2.2^R52A, R54A^ was found unchanged in respect to the wild type Ca_V_2.1 and Ca_V_2.2 respectively indicating that these proteins are not more stable or more highly expressed (data not shown, three independent experiments). The larger Ca_V_2.1^R57A, R59A^ current can be explained by the lack of tonic G-protein inhibition, as it has been shown that disrupting this motif completely abolished Ca_V_2.2 G-protein modulation (Canti et al., 1999). The truncated channels mimicking EA2 suppressed Ca_V_2.2^R52A, R54A^ and Ca_V_2.1^R57A, R59A^ trafficking and currents, in a similar manner to their effect on the wild-type channels. This indicates that disrupting the RAR motif in the full-length channel does not impede the deleterious interaction; in contrast, disrupting the same RAR motif present in the N-terminus of the truncated channels reduced their ability to produce dominant-negative suppression for both Ca_V_2.1 and Ca_V_2.2.

**Fig. 9:**
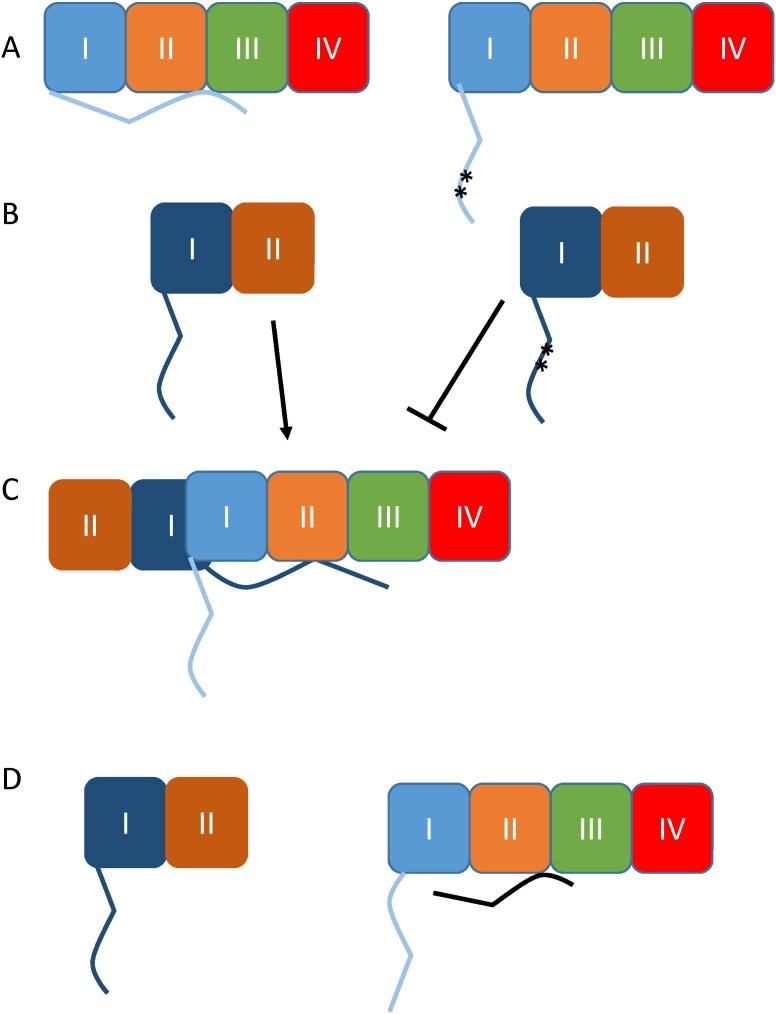
Diagram of the possible mechanism for involvement of the N-terminus in dominant-negative suppression. (A) The four domains of Ca_V_2 channels are shown in light blue, orange, green and red. The N-terminus is shown interacting with an intramolecular docking site on the left, whereas on the right interaction is reduced by mutation of the RAR motif (**). (B) The two domains of the truncated constructs are shown in dark blue and brown. On the right the truncated construct has a mutated RAR (**). (C) The N-terminus of the truncated construct can interact with the docking site on the full-length channel, initiating aggregation. This interaction is reduced by mutation of the RAR motif. (D) The free N-terminus interacts with the docking site on the full-length channel reducing the ability of the truncated channel to interact with this site.

We postulate that the N-terminus is normally involved in intramolecular docking with another part of the same channel, to form correctly folded and functional channels, and the RAR motif is involved in this interaction (Fig. 9A). This intramolecular docking is also involved in G-protein–mediated inhibition of the Ca_V_2 channels (Page et al., 1998, Canti et al., 1999). We propose that in the truncated channels this intramolecular interaction cannot occur, because the channel structure is incomplete and misfolded (Fig. 9B), with the consequence that the exposed N-terminus of the truncated channel is able to participate in competition for the N-terminal docking site on the full-length channel, to form a deleterious intermolecular interaction (Fig. 9B, C). When the RAR motif is mutated in the N-terminus of the truncated channels, it therefore reduces their ability to interact with docking site on the full length channel (Fig. 9B, C).

We have also shown that overexpression of an N-terminal peptide containing the RAR motif disrupted the deleterious interaction and interfered with the ability of the truncated domains to suppress trafficking and functional expression of the full-length channels (as represented in Fig. 9D). Although this effect was only partial in overexpression systems, strikingly, the same N-terminal peptide completely abolished the deleterious effect of the EA2 mutant on the endogenous P/Q-type current in DRG neurons. This discrepancy is probably due to the fact that the interaction occurs very early during protein synthesis in the endoplasmic reticulum (Page et al., 2004, Mezghrani et al., 2008). Thus the complex, comprising full-length and truncated channels, is formed co-translationally in expression systems, possibly involving additional sites as well as the N-terminus, and is retained in the endoplasmic reticulum with limited access to competition for its binding site by the free N-terminal construct. In contrast in DRG neurons the free N-terminus is probably expressed in excess compared to the endogenous channels, and better able to compete with EA2 for the N-terminal docking site on the full length channel. When the N-terminal RAR motif in the full-length channel is mutated to AAA, we propose that the intramolecular interaction of the N-terminus with its own docking site may be weakened (Fig 9A). This would agree with our finding that the free N-terminus was not able to disrupt the deleterious interaction between Ca_V_2.1^R57A, R59A^ and the EA2 mutant, and may indicate that the interaction is more robust and thus more difficult to disrupt.

The intramolecular docking mechanism we propose for Ca_V_2 is reminiscent of the role of the tetramerization domain (T1 domain) in the N-termini of Kv1–Kv4 potassium channels, which has been shown to be involved with the folding and oligomerization of the channels (Brueggemann et al., 2013). Oligomerization requires a threshold level of folding of the N-terminus and it has been suggested that this coupling between folding and assembly of Kv channels may be a common trait (Robinson and Deutsch, 2005). The interactions between T1 domains of Kv1.3 occur very early during protein synthesis in the endoplasmic reticulum, while the nascent peptides of different subunits are still attached to ribosomes (Lu et al., 2001).

In summary we have shown that uncoupling the deleterious interaction and thus releasing the full-length channel from its association with the truncated EA2 channel by expressing the key N-terminus residues partially restored channel functional expression in overexpression systems. More importantly the endogenous P/Q current in native neurons was fully recovered by expressing the same N-terminal peptide.

## Author contributions statement

A.C.D. and S.D. designed the experiments. S.D. and K.M.P. performed the experiments. K.M.P. and W.S.P. contributed to the molecular biology experiments. M.N.-R. and M.D. contributed to the DRG neuron dissociation, transfection and culture. S.D. analysed the data and S.D. and A.C.D. wrote the manuscript.

## Conflict of interest

The authors declare no competing financial interests.
